# Analysis of Genetic Diversity and Structure Pattern of *Indigofera Pseudotinctoria* in Karst Habitats of the Wushan Mountains Using AFLP Markers

**DOI:** 10.3390/molecules22101734

**Published:** 2017-10-16

**Authors:** Yan Fan, Chenglin Zhang, Wendan Wu, Wei He, Li Zhang, Xiao Ma

**Affiliations:** 1 Chongqing Academy of Animal Husbandry, Chongqing 400039, China; cq_fy001@163.com (Y.F.); heweili66@126.com (W.H.); zhangli10078244@163.com (L.Z.); 2 Department of Grassland Science, Animal Science and Technology College, Sichuan Agricultural University, Chengdu 611130, China; zcl0636@126.com (C.Z.); wuwendandan@163.com (W.W.)

**Keywords:** AFLP, genetic diversity, habitat fragmentation, *Indigofera pseudotinctoria*, structure pattern

## Abstract

*Indigofera pseudotinctoria* Mats is an agronomically and economically important perennial legume shrub with a high forage yield, protein content and strong adaptability, which is subject to natural habitat fragmentation and serious human disturbance. Until now, our knowledge of the genetic relationships and intraspecific genetic diversity for its wild collections is still poor, especially at small spatial scales. Here amplified fragment length polymorphism (AFLP) technology was employed for analysis of genetic diversity, differentiation, and structure of 364 genotypes of *I. pseudotinctoria* from 15 natural locations in Wushan Montain, a highly structured mountain with typical karst landforms in Southwest China. We also tested whether eco-climate factors has affected genetic structure by correlating genetic diversity with habitat features. A total of 515 distinctly scoreable bands were generated, and 324 of them were polymorphic. The polymorphic information content (PIC) ranged from 0.694 to 0.890 with an average of 0.789 per primer pair. On species level, Nei’s gene diversity (*H_j_*), the Bayesian genetic diversity index (*H_B_*) and the Shannon information index (*I*) were 0.2465, 0.2363 and 0.3772, respectively. The high differentiation among all sampling sites was detected (*F_ST_* = 0.2217, *G_ST_* = 0.1746, *G’_ST_* = 0.2060, *θ^B^* = 0.1844), and instead, gene flow among accessions (*N_m_* = 1.1819) was restricted. The population genetic structure resolved by the UPGMA tree, principal coordinate analysis, and Bayesian-based cluster analyses irrefutably grouped all accessions into two distinct clusters, i.e., lowland and highland groups. The population genetic structure resolved by the UPGMA tree, principal coordinate analysis, and Bayesian-based cluster analyses irrefutably grouped all accessions into two distinct clusters, i.e., lowland and highland groups. This structure pattern may indicate joint effects by the neutral evolution and natural selection. Restricted *N_m_* was observed across all accessions, and genetic barriers were detected between adjacent accessions due to specifically geographical landform.

## 1. Introduction

As a fundamental component of biodiversity, genetic diversity is indispensable to the population sustainability and evolution of plant species in changing environments. Its levels and patterns reflect the evolutionary history of the species (e.g., shifts in distribution, habitat fragmentation, population isolation, bottleneck effects and founder events) and the interactions of various processes, such as genetic drift, breeding system, selection and gene flow [1,2]. Consequently, reliable estimates of population differentiation may play a crucial role in understanding connectivity between populations and could be a prerequisite for conserving and using sources of wild germplasm [3]. Some studies on finer-scale spatial genetic structure have shown the importance of gene flow and its impact on genetic structure at a local scale [4]. Gene flow interruption is the primary step towards reproductive isolation under geographical isolation, thereby increasing interspecific differentiation and inbreeding while decreasing intraspecific variation [5]. However, at a small spatial scale, micro-geographical differentiation among local populations without discrete isolation is attributed more to species characteristics such as population size, breeding system and seed dispersal than to selection [6]. These neighboring populations may suffer from habitat fragmentation that increases spatial isolation, reduces population size and density, leads to strong divergence and decreases genetic diversity [7,8].

Although limitation of gene flow remains the main reason to spatial genetic structure, historical events, eco-geographic conditions and anthropic effects can also influence the spatial distribution and structure pattern of a species [9,10]. Typical of mountain ranges, elevation gradients are special cases of landscape variation that influence both genetic structure and diversity of populations, particularly those located at the upper and lower distributional limits [11]. Driven by the joint effects of natural selection and neutral evolution, ecological divergence could interfere the balance between genetic drift and gene flow across the landscape and alter genetic structure within a species [12,13]. If natural selection has affected the genome of a species, the outlier loci should show limited differentiation between populations in the case of balancing selection, or extensive differentiation if divergent selection has had an effect. Alternatively, if natural selection has not affected specific sites throughout the genome, there should be no evidence of outlying loci [14]. Therefore, analysis across multiple populations allows separation of demographic and environmental factors in determining population structure of species, and infers the primarily diverged force due to selectively neutral evolution or natural selection [15].

Karsts represent distinctive landforms and hydrological systems on Earth. Karst regions are extensive in southern China and have generated the extraordinary floras present on these landscapes [16,17]. The high edaphic and topographic heterogeneity of karst landscapes offers a multitude of ecological niches for plants to diversify and specialize; accordingly, more than 4287 plant species have been found in karst areas in southern China, of which approximately 28% are karst endemics [18]. However, karst ecosystems are extremely fragile eco-geo-environments. The high dissolution rate of soluble and porous bedrock (e.g., carbonate rocks, limestone) makes the soil alkaline and barren with a double-layered structure, creating conditions of low soil water content and periodic water deficiency, which are harmful to the growth and reproduction of plant communities [19]. In addition, due to topographic heterogeneity (e.g., deep canyons, caves and rivers formed by geological events) and global warming, many endemic plants in karst areas tend to suffer from fragmentation of suitable habitats and reduction in species diversity [20,21]. Furthermore, the unique biota in karst areas has been sharply affected by recent human activities, such as large-scale logging, agricultural expansion, livestock overgrazing and fire, which have led to extensive water and soil erosion and serious land degradation in the form of rocky desertification, reducing the stability of the karst ecosystem [22,23]. Under increasing habitat pressure caused by both human disturbance and global climate change, many plant communities in the karst area have become rare or endangered, especially species endemic to the area [16]. However, little is known about naturally heterogeneous landscape affecting genetic diversity and structure in endemic plants to karst mountains in southern China, especially at fine spatial scales. The information on the distribution of genetic variation over the scale of small geographical regions is crucial to our understanding of the ecological adaptation of any endemic species.

*Indigofera L.*, a large pantropical genus belonging to the Fabaceae, contains 750 species worldwide and occurs on all major land masses, most abundantly in Africa and Asia [24,25]. Among these, *Indigofera pseudotinctoria* Mats (false indigo), a perennial diploid shrub (2*n* = 2*x* = 16) [26], is an economically and agronomically important species distributed in China, Japan, and Korea, occurring sparsely in grasslands, hillsides and shrublands at elevations ranging from 100 m to 2000 m [27]. Because of its strong nitrogen fixation and adaptation capacity as well as its various organic compounds and mineral elements, it serves as medicine and animal feed and plays an important role in ecological protection in southern China, especially in water and soil conservation, by providing slope stability in karst areas [28,29]. Although this plant can effectively resist environmental degradation in karst landscapes, it suffers from a fragmented distribution caused by human disturbance and climate change; the population size and density of *I. pseudotinctoria* have shown a continuous and rapid decrease, even threatening the karst ecosystem [30]. Therefore, a good knowledge of its habitat factors as well as the genetic diversity and structure of native *I. pseudotinctoria* populations will provide a better understanding of the relevant ecological and genetic processes, which is a necessary prerequisite for both ecological restoration and propagation of the remnant populations in the karst areas of southern China [31].

The amplified fragment length polymorphism technique (AFLP) is a high resolution PCR-based, multi-locus molecular marker assay with high reproducibility with no requirement for prior sequence information of the genome being studied [32]. It is capable of generating a large number of reproducible fragments with genome wide distribution [3,32]. Due to high efficiency and reproducibility of AFLP, it has been widely used to assess genetic relationships and intraspecific genetic diversity of wild or natural plant populations [5,33]. To date, no AFLP marker has been used to assess the genetic diversity of native *I. pseudotinctoria* populations of China. The objective of present study were to estimate genetic structure and diversity in local *I. pseudotinctoria* accessions in Wushan Mountain of southern China, among which all collection sites could represent different eco-climatic conditions. Since increased environmental variability on a small geographical scale favors genetic differentiation influenced by differential local adaptation [4,5,34], our hypothesis was that genetic accession structure would exist within *I. pseudotinctoria* collections from Wushan Mountain in Southwest China, and that genetic variability would be associated with certain environmental factors (altitude, annual mean temperature and annual precipitation, etc). This information may be useful to elaborate the relationship between local adaptation and genetic diversity as well as make the desirably managing strategies of mesquite resource.

## 2. Results

### 2.1. Gene Diversity of I. Pseudotinctoria

At species level, amplification and polymorphism of the six AFLP primer pairs were showed in Table 1. A total of 515 AFLP bands were clearly visible in 364 individuals, with an average of 85.83 bands generated by each pair of primers. Among all the bands, 324 (62.91%) were polymorphic. The number of polymorphic bands ranged from 49 for primer pair E59M55 to 57 for E76M85 and E85M60, while the percentage of polymorphic bands varied from 54.90% for primer pair E53M55 to 70.37% for E76M85. In addition, the polymorphic information content (PIC) for each primer pair ranged from 0.1690 to 0.3029, with an average of 0.2243. The Nei’s gene diversity (*H_j_*) of primer was between 0.2308 for primer pair E53M55 and 0.3980 for E76M85. The Shannon information index (*I*) of primer was also computed, and the results varied from 0.1998 to 0.3189 with a mean of 0.2774.

The parameters of genetic diversity of 15 accessions are shown in Table 2. The highest number of polymorphic loci (*N_p_*) was 296 in ace.E, with 91.36% polymorphic loci, while the lowest *N_p_* was 202 in popO, with 62.35% polymorphic loci. A linkage disequilibrium (LD) analysis for each accession was conducted that the percentage of LD ranged from 9.01% in acc.B to 12.72% in acc.F, while the total rate of LD was 10.73%. This result revealed relatively low recombination and replacement. The observed number of alleles per locus (*N_a_*) ranged from 1.6235 to 1.9136, and the effective number of alleles per locus (*N_e_*) varied from 1.2874 in acc.F to 1.4491 in acc.B, with average values of 1.7757 and 1.3661, respectively. Different genetic diversity indexes were calculated and showed high genetic diversity among accessions. Nei’s gene diversity index (*H_j_*) ranged from 0.2020 in acc.F to 0.2992 in acc.B, the overall *H_j_* was 0.2986, and the average was 0.2465. Similarly, the Shannon information index (*I*) found the highest diversity within acc.B (0.4130) and the lowest within acc.F (0.2708); the total *H_o_* was 0.4291, and the mean was 0.3407. In contrast, the Bayesian gene diversity index (*H_B_*) revealed the highest diversity within acc.A (0.2869), although the lowest diversity was still found within acc.F (0.1993), and the total and average of *H_B_* were 0.2895 and 0.2362, respectively.

### 2.2. Structure Pattern of I. Pseudotinctoria

**Figure 1:**
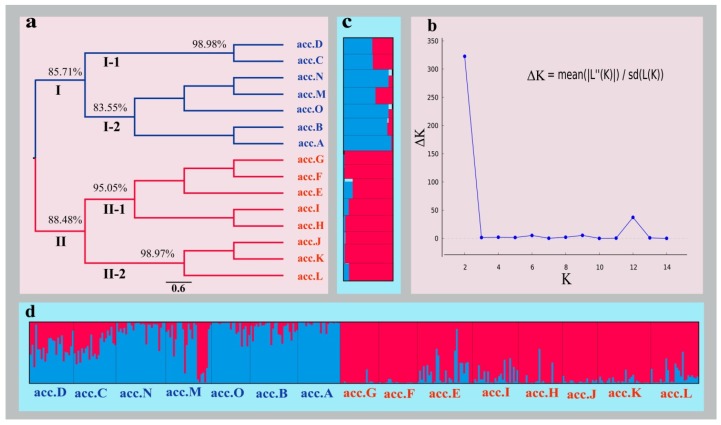
Cluster analysis of *I. pseudotinctoria* ((**a**) unweighted pair-group method with arithmetic means) tree for 15 accessions; (**b**) the relationship between K and ΔK (Delta K); (**c**) STRUCTURE analysis for 15 accessions when K = 2; (**d**) STRUCTURE analysis for 364 individuals when K = 2). Two colors represented differently potential genetic compositions.

For studying the genetic structure of *I. pseudotinctoria*, an unweighted pair-group method with arithmetic mean (UPGMA) clustering analysis was conducted to estimate the genetic relationships among the 15 accessions using Nei’s genetic distance matrix (Figure 1a). The result showed that these accessions were classified into two main clusters, which was confirmed by Bayesian model-based cluster analysis with a clear peak value of the ΔK statistic (Figure 1b) at K = 2. In addition, STRUCTURE plots were created for 15 accessions (Figure 1c) and 364 individuals (Figure 1d) at K = 2. The cluster analysis (UPGMA tree and STRUCTURE analysis) strongly suggested that these accessions gathered into clusters based on similarity of elevation. The Cluster I contained acc.A, acc.B, acc.C, acc.D, acc.M, acc.N, and acc.O, which were situated at 311~913 m. The Cluster II contained the remaining eight accessions, from acc.E to acc.L, which were situated at 1002~1612 m. Moreover, each cluster could be divided into two sub-clusters. The first sub-cluster belonging to Cluster I contained acc.C and acc.D, and the second sub-cluster of Cluster I contained acc.A, acc.B, acc.M, acc.N and acc.O. In Cluster II, acc.E to acc.I and acc.J to acc.L represented first sub-cluster and second sub-cluster, respectively. Overall, the cluster analysis demonstrated that 15 accessions gathered into two clusters that could be approximated by elevation.

**Figure 2:**
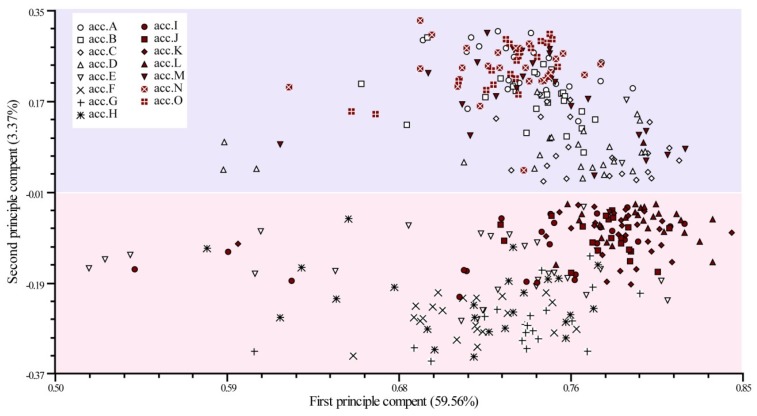
Principle coordinate analysis plot based on AFLP makers for 364 individuals from 15 accessions. The individuals of per accession were represented by the same shapes.

To further investigate accession structure, principal coordinate analysis (PCoA) was performed on the entire dataset of 324 genotypes (Figure 2). PCoA based on a similarity matrix explained 59.56% and 3.37% of the genetic variation with the first and the second PCoA axes, respectively. This result revealed that these individuals could basically be divided into two groups. The individuals from acc.A, acc.B, acc.C, acc.D, acc.M, acc.N, and acc.O gathered together in the upper part of the plot. The individuals from acc.E to acc.L mainly gathered at the lower part of the plot. This result confirms that of the cluster analysis. Furthermore, the individuals within each of acc.A, acc.B, acc.F, acc.G, and acc.K gathered relatively closely, and this result was also visualized in the STRUCTURE analysis. 

### 2.3. Genetic Differentiation and Gene Flow

For estimating the partitioning of genetic variance within *I. pseudotinctoria*, an analysis of molecular variance (AMOVA) was conducted (Table 3). The results attributed 22.17% of the variance to differences among accessions, while 77.83% could be attributed to variability within accessions. Based on cluster analysis, we divided the 15 accessions into two Clusters according their elevation. The accessions situated at 311~913 m were considered the lowland Cluster (corresponding to Cluster I in UPGMA tree and STRUCTURE analysis), and the accessions at elevations of 1002~1612 m were considered the highland Cluster (corresponding to Cluster II in UPGMA tree and STRUCTURE analysis). Then, AMOVA was calculated for the two Clusters, which showed that 8.98% of the genetic variance occurred between the two classified Clusters, while 25.43% of the genetic variance occurred within accessions.

To further study the variance of *I. pseudotinctoria* at a small scale, a variety of genetic differentiation coefficients were calculated (Table 4). 

Nei’s genetic differentiation coefficient (*G_ST_*) was 0.1746, indicating that 17.46% of the genetic variance occurred among the 15 accessions. Shannon’s coefficient of genetic differentiation (*G’_ST_*) estimated that 20.60% of the variance was detected among all accessions. Moreover, accession diversity was computed for the two groups partitioned by elevation, revealing significantly higher average within-accession diversity of the lowland group (*H_S_* = 0.2641, *H_pop_* = 0.3618) than the highland group (*H_S_* = 0.2142, *H_pop_* = 0.3223), according to significance testing in SPSS software using a nonparametric test (*p* < 0.05). In addition, genetic differentiation within the lowland group (*G_ST_* = 0.1255, *G’_ST_* = 0.1789, *F_ST_* = 0.2124) was higher than that within the highland group (*G_ST_* = 0.1015, *G’_ST_* = 0.1511, *F_ST_* = 0.1491). For AFLP markers, the results of *F_ST_* estimated by Bayesian analysis are listed in Table 5. The smallest mean deviance information criterion (DIC), 19,375.6, was obtained under the f = 0 model, suggesting that the *f* = 0 model was more suitable than other models for this dataset. The mean *θ^B^* (analogous to *F_ST_*) in the *f* = 0 model was 0.1844 (standard deviation (SD) = 0.0022) among accessions, and the magnitude of genetic differentiation was analogous to the above coefficients.

**Figure 3:**
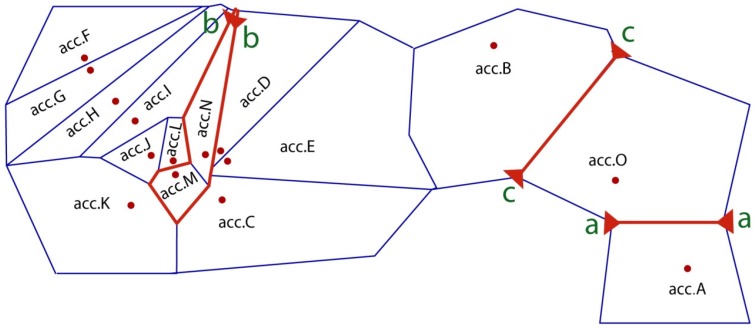
Genetic barriers predicted by BARRIER software (version 2.2, Syracuse University, Syracuse, NY, USA). Lines a, b, and c indicated genetic barriers.

In addition, gene flow (*N_m_*) was calculated to be 1.1819 individuals per generation between accessions (Table 4). Further, a genetic barrier prediction analysis using Monmonier’s maximum difference algorithm revealed three likely barriers to gene flow (Figure 3). The first barrier (aa) isolated the acc.A site from the rest of the collection sites, the second (bb) separated acc.M and acc.N from the neighboring accessions, and the third (cc) was predicted to isolate the acc.O site from acc.B. Moreover, we have calculated the pairwise *F_ST_* and *N_m_* for 15 *I. pseudotinctoria* accessions (Table 6). It revealed that the highest genetic differentiation (*F_ST_* = 0.2730) and lowest gene flow (*N_m_* = 0.6658) were between acc.A and acc.F. According to above barrier analysis, the *F_ST_* and *N_m_* between acc.A and acc.O were 0.2176 and 0.8989, respectively.

### 2.4. Genetic Diversity Associated with Environmental Factors

**Figure 4:**
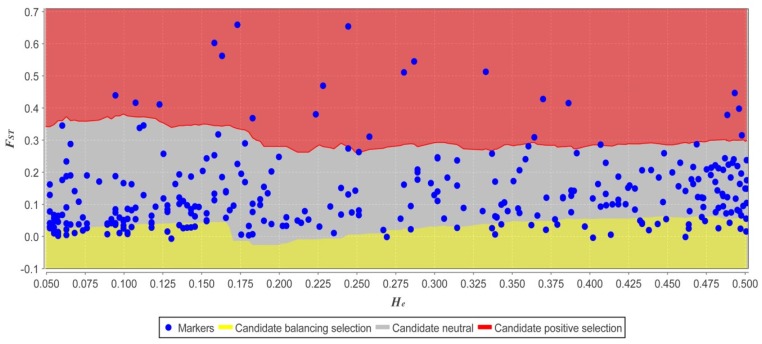
Distribution of *F_ST_* values as a function of heterozygosity for interaccessional comparisons. Each dot represents an AFLP locus. The dots in red board are classified as outliers potentially under divergent selection. The dots are classified as neutral loci in gray board and balancing loci in yellow board.

Among the 324 AFLP loci, the *F_ST_* values of 21 loci (6.48%) were significantly high deviating from 95% confidence intervals In Mcheza program, thus the 21 loci were potential outliers under divergent selection (Figure 4). A spatial autocorrelation analysis was conducted and revealed very slight interactions among accessions (*r* = 0.146, *p* < 0.01). The Mantel tests showed that [*F_ST_*/(1 − *F_ST_*)] significantly decreased with increasing geographic altitude (*r* = −0.755, *p* < 0.01), indicating a pattern of lower genetic diversity at higher altitudes. The [*F_ST_*/(1 − *F_ST_*)] was not correlated to annual precipitation (*r* = −0.343, *p* < 0.01) and annual mean temperature (*r* = −0.4423, *p* < 0.01).

## 3. Discussion

### 3.1. Genetic Diversity Across all Accessions

Genetic diversity is a vital basis for species studies because its amount and distribution are likely to affect the evolutionary potential of species or populations, which can include expanding distribution range and adapting to habitat [35]. Otao et al. [27] have researched the genetic diversity of three *I. pseudotinctoria* populations comprising two native populations in Japan and one population transported from China using 14 microsatellite primers and found no LD in any of the three studied populations, while 10.73% LD was detected in our study. Theoretical studies have noted that many interrelated factors are involved in the formation of patterns of LD, including genetic drift, population admixture, and natural selection [36,37]. The heterozygosity (*H_e_* = 0.346) and effective number of alleles per locus (*N_e_* = 3.310) found by Otao et al. were higher than in our study (*H_j_* = 0.2986, *N_e_* = 1.4433). These differences are due to inherent differences between the AFLP and microsatellite DNA (simple sequence repeat, SSR) techniques because SSR markers are co-dominant, whereas AFLP is dominant [38].

Most of the genetic diversity of *I. pseudotinctoria* is distributed within accessions (77.83%). This high level of genetic diversity within accessions may be because *I. pseudotinctoria* is an outcrossing perennial species; such species usually contain higher genetic diversity than that in autogamous annual species, as shown in other studies [39,40,41]. The pattern of genetic diversity found in *I. pseudotinctoria* most likely reflected its ecological and life history traits; that is, historical fragmentation derived from geological movement and subsequent climatic oscillation would hinder gene flow within accessions. A high-longevity species rather than losing genetic diversity could maintain genetic diversity over a longer period [42,43]. For the interpretation of *F_ST_*, it has been suggested that a value lying in the range a value between 0.15 and 0.25 meant great differentiation so our study has revealed high genetic differentiation among accessions (*F_ST_* = 0.2217). This explanation might be the small size and fragmented distribution of *I. pseudotinctoria* accessions due to heterogeneous environment and hunman activities. So gene flow among accessions is limited and genetic drift might lead to the loss of genetic diversity and increase of genetic differentiation for some accessions, especially for those accessions with small size [44,45].

### 3.2. Genetic Diversity Related to Geographic and Climate Parameters

Autocorrelation, derived from the first law of geography, is a very general property of ecological variables along a time series (temporal autocorrelation) or across geographic space (spatial autocorrelation) [46], and it has been used in plant in situ conservation. According to spatial autocorrelation analysis, populations that are physically closer together are more easily affected by each other with respect to intra-population diversity and inter-population variance [47]. In this study, the spatial autocorrelation analysis was conducted with Moran’s Index, revealing very slight interactions among *I. pseudotinctoria* accessions (*r* = 0.146, *p* < 0.01); thus, all the accessions were suitable, and all of them were used for calculations of genetics with no bias.

Remarkable variation occurs in the distribution patterns of genetic population diversity along elevation gradients, and greater genetic diversity is generally found within lowland populations [48,49]; however, genetic diversity peaks on higher slopes are also observed. In this study, a regression analysis between [*F_ST_*/(1 − *F_ST_*)] and geographic altitude revealed a strong and significant negative correlation (*r* = 0.755, *p* < 0.01) between within-accession diversity and site elevation, i.e., lower diversity at highland sites and higher diversity at lowland sites. As an insect-pollinated plant species, *I. pseudotinctoria* is thought to breed by xenogamy, which requires pollinators for outbreeding [25]. Schuster et al. [12] have found that large-scale elevation gradients represented significant barriers to the movement of pollinators and seed dispersers, limiting gene flow and further aggravating the divergence among upper- and lower-elevation *Podocarpus parlatorei* populations [50]. The empirical research revealed that lower size, density and visiting frequency of pollinators at higher altitudes [49]; in other words, *I. pseudotinctoria* accessions at the upper distributional range would have less opportunity for allelic exchange than lowland accessions that exhibit less genetic diversity. Because the ecological isolation of *I. pseudotinctoria* in karst areas driven by habitat heterogeneity and fragmentation could hinder gene flow among accessions and cause genetic erosion by genetic drift, local adaptation and inbreeding [50,51].

Climate factors such as temperature and precipitation can strongly influence population diversity and structure for a species [9]. A slight correlation was found between [*F_ST_*/(1 − *F_ST_*)] and annual mean temperature (*r* = 0.250, *p* < 0.01) in this study. In fact, temperatures decline along with rising elevation gradients; thus, the extremely low temperature at peaks can threaten local life, decreasing population diversity and size and even resulting in extinction [42]. This is a potential reason to explain the lower genetic diversity in higher altitudes. Because of the difference of precipitation among these sampling sites was very little, so that the annual precipitation was not a factor affecting accession diversity of *I. pseudotinctoria* at this small spatial scale (*r* = 0.050, *p* < 0.01).

### 3.3. Structure Pattern and Gene Flow

Generally, outcrossing species have higher levels of gene flow (*N_m_*) that can resist the effect of genetic drift within populations and prevent the differentiation of populations [52]. When the value of *N_m_* is greater than 1, this resistance is stronger; when the value of *N_m_* is less than 1, genetic drift can lead to genetic differentiation among populations [53]. In this study, *N_m_* was 1.1819 (*p* < 0.01) among all sampling accessions, indicating a small magnitude of gene flow among the accessions. This restricted gene flow reflected that the natural landforms in the sampling area led to ecological isolation, which reduced gene flow and increased genetic divergence among the accessions [43]. Reductions in gene flow between accessions due to divergent selection can also enhance the impact of selectively neutral mechanisms of evolution (e.g., genetic drift), further accelerating differentiation between accessions [54,55]. So some genetic differentiation was found between lowland group and highland group (Table 4: *F_CT_* = 0.0898). The gene flow (*N_m_*) was calculated for two groups in different elevations Based on *F_ST_*. We found that gene flow within lowland group (*N_m_* = 1.7421) and highland group (*N_m_* = 2.2128) were both high, suggesting that gene flow resisted intra-accession drift and hindered inter-accession differentiation. However, the lower level of *N_m_* among the lowland accessions was not consistent with the lower frequency of pollinators at higher altitudes [9], suggesting that the pollinator difference derived from the elevation gradient was not the main factor affecting gene flow in lowland and highland accessions in the present study, but rather the divergent evolution by natural selection under specially geographic landforms. It was demonstrated in divergent outlier analysis that 21 loci were selected under natural evolution. As a preferred leguminous feed for livestock, especially goats, *I. pseudotinctoria* in lowland sites was sharply disturbed by local animal grazing because these livestock directly change the accession size and distribution by ingestion and spreading the indigestible seeds [56]. In addition, human activities, such as deforestation and urban expansion also partly change the community structure, complexity and heterogeneity and increase the fragmentation of the *I. pseudotinctoria* in different elevation gradients [18].

The genetic BARRIER analysis using Monmonier’s maximum difference algorithm [57] revealed a highly significant genetic barrier (aa) between acc.A and acc.B, and then we calculated the relevant coefficients *G_ST_* and *N_m_*, which indicated high genetic differentiation (*G_ST_* = 0.3162, *p* < 0.01) and very low gene flow (*N_m_* = 0.5406) between them. The second genetic barrier (bb) isolated acc.M and acc.N from the adjacent accessions, resulting in high genetic differentiation (*G_ST_* = 0.2498, *p* < 0.01) and low gene flow (*N_m_* = 0.7508). A third genetic barrier occurred between acc.B and acc.O, and similar results were observed (*G_ST_* = 0.2184, *N_m_* = 0.8947). Although the distance between adjacent accessions is not great, gene flow has been seriously restricted by genetic barriers derived from habitat fragmentation; for example, acc.M and acc.N are located in deep canyons surrounded by high mountains, while acc.O is separated from acc.A and acc.B by the wide Yangtze River. These ecological barriers have promoted divergence between adjacent accessions by the joint effects of neutral evolution and natural selection and even led to reproductive isolation in future generations.

## 4. Materials and Methods

### 4.1. Study Sites and Sampling

**Figure 5:**
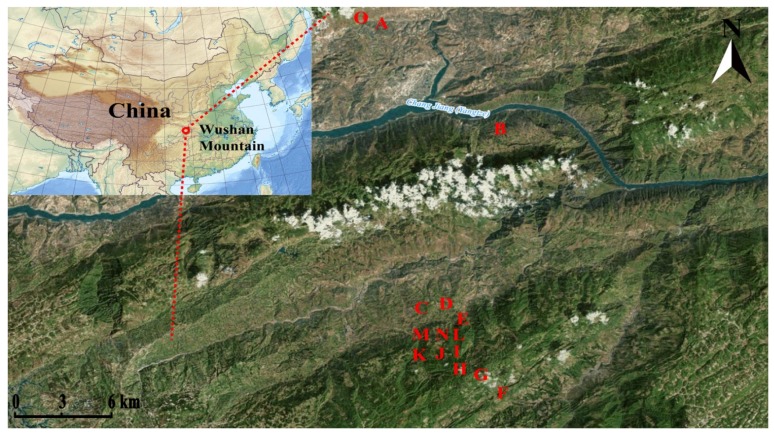
The sampling sites of 15 *I. pseudotinctoria* accessions in Wushan Mountains at Southwest China.

Wushan Mountain is located in the northeast of China's Chongqing Municipality, flowed across by the Yangtze River and its several branches. It has a subtropical monsoon climate and the typical karst landform of south China, filled with many deep valleys, sharp cliffs and slopes. The elevation drop from top to bottom is over 2000 m, and the various vegetation types significantly change along with the increase of elevation [58]. We analyzed 364 adult individuals of *Indigofera pseudotinctoria* collected from 15 native accessions located in Wushan Mountain (Table 7). The sampling area ranged from acc.O (109°51’13.92” E) in the east to acc.B (109°55’40.15” E) in the west and from acc.O (31°07’3.41” N) in the north to acc.F (30°54’50.84” N) in the south (Figure 5). The annual mean temperature and annual precipitation in the sampling area were approximately 15.04 °C and 1124.5 mm, respectively. The sampling area was montane karst, and the soils were loess soil and purple soil, mostly alkaline, with soil organic matter varying from 6.2 g/kg in the acc.A site to 66.3 g/kg in the acc.F site. Between 19 and 30 individuals were collected for each accession, and the geographical coordinates of each accession were recorded using a global positioning system (GPS) device. For genetic analysis, samples from each individual consisted of 5–10 young leaves that were preserved in zip-lock plastic bags containing silica gel and stored at −20 °C for DNA isolation. 

### 4.2. DNA Isolation

The leaves were rinsed with 70% ethanol, dried and then ground to a fine powder under liquid nitrogen using a mortar and pestle. Genomic DNA was isolated from 200 mg of the ground leaf tissue from each sample using a modified CTAB method [59]. The quality and quantity of genomic DNA were verified by 0.8% (*w*/*v*) agarose gel electrophoresis in 1X TBE buffer (0.09 M Tris–borate, 2 mM EDTA, pH 8.3), run 45 min at 100 V, stained with ethidium bromide and visualized under UV light. The DNA obtained was stored at −20 °C in TE buffer (Tris–EDTA) until use.

### 4.3. AFLP Procedure

AFLP reactions were performed as described by Vuylsteke et al. [32] with minor modifications. Six selective pairs of MseI (M-GACGATGAGTCCTGAG) and EcoRI (E-CTCGTAGACTGCGTACC) primers were used in the selective amplification, having been identified as the most informative and reliable from a set of 80 primer pairs during a preliminary investigation. Amplification products were purified with a spin column purification kit for PCR products as following: 1 PCR buffer, 2 mM MgCl_2_, 2 mM dNTP, 40 ng of each of EcoRI primer and MseI primer, 1 U Taq polymerase, and 5.0 µL pre-amplified template DNA. The fragments amplified in the latter step were subjected to capillary electrophoresis using an ABI 3500 (Applied Biosystems, Foster City, CA, USA), and the fluorescent fragments of each sample were treated using GeneMarker (version 2.6, SoftGenetics, State College, PA, USA). In order to evaluate the reproducibility of six AFLP primer pairs selected by the pre-test experiment, we compared the profiles from five replicates for 10 randomly chosen plants from all accessions, which yielded the average reproducibility of 96.8%. Non-reproducible markers were not taken into consideration.

### 4.4. Data Analysis

#### 4.4.1. Band Statistics

The fluorescent AFLP patterns were visualized as electropherograms, in which each peak of fluorescence corresponded to an amplified DNA fragment. Each AFLP band between 80 and 500 bp was considered a single biallelic locus with an amplifiable (1) and a null (0) allele to generate AFLP binary matrices, then an integrated binary matrix of AFLP phenotypes was constructed for further analysis.

#### 4.4.2. Gene Diversity

Linkage disequilibrium (LD) was calculated using ARLEQUIN version3.0 [60] to detect whether recent accession bottlenecks and extinction-replacement had occurred at a significance level of 0.05. Then, the following genetic diversity parameters were calculated for each primer set: the total number of bands (TNB), polymorphic bands (PB), polymorphism rate (P) and polymorphic information content (PIC). Parameters were calculated using Excel 2013 software using the formula: PIC = 1 − ∑P^2^_ij_, where P_ij_ is the frequency of the *j*th allele for the *i*th AFLP locus. Genetic diversity (*H_j_*) and Shannon information index (*I*) for primers were calculated in POPGENE (version 1.32, University of Alberta, Edmonton, AB, Canada) [61]. At the species level, the number of polymorphic loci (*N_p_*), percentage of polymorphic loci (*PLP*), observed number of alleles per locus (*N_a_*), effective number of alleles per locus (*N_e_*), and Shannon information index (*I*) with its standard error were calculated using POPGENE version 1.32 [61]. Nei’s gene diversity (*H_j_*) with its standard error was computed using AFLP-SURV version 1.0 [61] using the approach of Lynch and Milligan and the Bayesian method with non-uniform prior distribution to calculate allelic frequencies assuming Hardy-Weinberg equilibrium [62]. Nevertheless, taking into account a possible bias caused by this assumption, Bayesian gene diversity (*H_B_*) and its standard error were also calculated using Hickory version 1.1 [63], which does not assume Hardy-Weinberg equilibrium within accessions.

#### 4.4.3. Genetic Structure

To further research the genetic relationship among 15 accessions, Nei’s genetic distance (GD) matrix of 15 accessions was calculated with a Bayesian method using AFLP-SURV version 1.0 [61] with non-uniform distribution and 10,000 bootstrapping replicates, and then the results were used as inputs for an UPGMA clustering analysis using the CONSENSE module in the PHYLIP software package, version 3.69 [64]. Meanwhile, a PCoA for 364 individuals was computed using R package “stats” [65] to ordinate the spatial relationships among the individuals. Moreover, Bayesian model-based cluster analysis was performed to infer genetic structure and to define the number of clusters in the dataset using the software STRUCTURE, version 2.3.4 [33]. Correlated allele frequencies and an admixed model were applied with a burn-in period of 50,000 and 100,000 MCMC replicates after burn-in. The range of cluster number (K) was predefined from 1 to 14 with 10 runs for each K. The optimum value of K was determined by examination of the ΔKs and L(K) using STRUCTURE HARVESTER (version 0.6.94, UCSC, Santa cruz, CA, USA) [66]; these metrics usually plateaued or increased slightly after the “optimum K” was reached. A consensus STRUCTURE plot was obtained from the admixture repeats using the greedy algorithm in CLUMPP version 1.1 [67], and final plots were visualized in STRUCTURE PLOT [68]. 

#### 4.4.4. Differentiation and Gene Flow

To further study the magnitude of genetic differentiation (*F_ST_*), the partitioning of variation at different levels was calculated by AMOVA using “vegan” package [5] in R program with 1000 permutations. Additionally, Nei’s coefficient of genetic differentiation (*G_ST_*) was calculated using AFLP-SURV version 1.0 [61] with 10,000 permutations according to the formula: *G_ST_* = (*H_T_* − *H_S_*)/*H_T_*, in which *H_T_* and *H_S_* were total Nei’s gene diversity and average Nei’s gene diversity within accessions, respectively. Furthermore, Shannon’s coefficient of genetic differentiation (*G’_ST_*) was also estimated as *G’_ST_* = (*I_sp_* − *I_pop_*)/*I_sp_*, in which *I_sp_* was total Shannon diversity and *I_pop_* was average Shannon diversity within accessions. Bayes posteriors were obtained for a full model (*f*, inbreeding coefficient, and *θ*, accession differentiation, were estimated), a model in which *f* was set to 0, a model in which *θ^B^* was set to 0 and an *f*-free model (estimates *θ^B^* without estimating *f*) using Hickory version 1.10 [69]. The DIC was used to estimate the fit of the different models, and those with lower DIC values and differences no larger than 6 units between them were chosen. According to Nei’s genetic differentiation (*G_ST_*), gene flow *N_m_* was estimated following the method: *N_m_* = (1 − *G_ST_*)/4*G_ST_*; *N_m_* allows accessions to resist the effect of genetic drift and prevents the differentiation of accessions for *N_m_* > 1; for *N_m_* < 1, genetic drift can lead to genetic differentiation among accessions [70].

#### 4.4.5. Genetic Barriers and Divergence Outlier Analysis

A specific test was devised to suggest historical barriers to gene flow among collection sites using the program BARRIER (version 2.2, Syracuse University, NY, USA) [57], according to Monmonier’s maximum difference algorithm with the geographic coordinates of each sampling site and the Nei’s GD calculated in AFLP-SURV as inputs. For detecting loci under selection, divergence outlier analysis (DOA) was conducted using the Mcheza program [71]. Pairwise comparison of *F_ST_* values between the accessions was conducted with default settings. The expected *F_ST_* distribution under the null hypothesis of neutrality was calculated with 50,000 simulations and a false discovery rate of 0.1 within the 95% confidence interval in 10 different simulations. As calculation of the initial mean *F_ST_* found in the dataset includes all potentially selected loci (i.e., loci falling outside of the confidence interval), those loci were removed from calculation of the “neutral” mean *F_ST_* [72]. The mean simulated *F_ST_* was forced to approximate the initial mean *F_ST_* by application of a bisection algorithm over repeated simulations.

#### 4.4.6. Correlation Analysis

A spatial autocorrelation analysis was conducted using SAM version 4.0 [73] to detect interrelation among minimum units obeying Moran’s Index. Then, a regression analyses between [*F_ST_*/(1 − *F_ST_*)] and environment parameters were calculated using “ade4” package [74] in R program with 95% confidence level.

## 5. Conclusions

The results of this study demonstrate that environmental conditions in different habitats are a major factor influencing the hereditary characteristics of *I. pseudotinctoria*. The gene diversity of *I. pseudotinctoria* was high and decreased with increasing elevation. The accessions from lowland and highland areas clustered into separate groups and the structure pattern of *I. pseudotinctoria* was affected by the joint effects of neutral evolution and natural selection. Higher gene diversity and differentiation occurred in lowland accessions than in highland accessions. Restricted gene flow was observed at different spatial scales, whereas genetic drift was detected in adjacent accessions due to geographic barriers. Suffering from the degeneration due to climatic changing and human activities, the accession size and density of *I. pseudotinctoria* gradually reduce, thus we should protect the local environment for maintaining the diversity of *I. pseudotinctoria.* Furthermore, the very high proportion of genetic diversity residing within locations encourages the intensive selection and collection of elite genotypes from each location for the genetic improvement of *I. pseudotinctoria*.

## Figures and Tables

**Table 1:** Amplification and polymorphism of the PCR products obtained with 6 AFLP primer pairs. Total number of bands (TNB), number of polymorphic bands (NPB), percentage of polymorphic bands (PPB), polymorphic information content (PIC), Nei’s gene diversity (*H_j_*), Shannon information index (*I*).

Primer	TNB	NPB	PPB(%)	PIC	*H_j_*	*H_o_*
E53M55	102	56	54.90%	0.1690	0.2308	0.3189
E59M55	80	49	61.25%	0.3029	0.2773	0.1998
E76M85	81	57	70.37%	0.2481	0.3980	0.2635
E78M85	80	54	67.50%	0.2341	0.3863	0.2829
E85M60	84	57	67.86%	0.2211	0.3308	0.2826
E86M85	88	51	57.95%	0.1708	0.2949	0.3165
Total	515	324	62.91%	0.2243	0.4023	0.3764
Mean	85.83	54	62.91%	0.2243	0.3197	0.2774

**Table 2:** Genetic diversity of 15 *Indigofera pseudotinctoria* accessions. Accession name (Acc.), number of polymorphic loci (*N_p_*), percentage of polymorphic loci (*PLP*), percentage of linkage disequilibrium at the 5% level (LD), observed number of alleles per locus (*N_a_*), effective number of alleles per locus (*N_e_*), Nei’s gene diversity index (*H_j_*), Shannon information index (*I*), and Bayesian gene diversity index (*H_B_*).

Acc.	*N_p_*	*PLP*(%)	LD(%)	*N_a_*	*N_e_*	*H_j_*	*H_o_*	*H_B_*
A	253	78.09	10.65	1.7809	1.4478	0.2985 ± 0.0097	0.3907 ± 0.0205	0.2869 ± 0.0032
B	282	80.74	9.01	1.8704	1.4491	0.2992 ± 0.0090	0.4130 ± 0.0217	0.2816 ± 0.0031
C	286	88.27	12.41	1.8827	1.4036	0.2779 ± 0.0092	0.3788 ± 0.0199	0.2580 ± 0.0032
D	293	90.43	5.74	1.8889	1.4304	0.2805 ± 0.0097	0.3966 ± 0.0208	0.2605 ± 0.0027
E	296	91.36	12.52	1.9136	1.4086	0.2563 ± 0.0100	0.3818 ± 0.0200	0.2482 ± 0.0026
F	202	62.35	12.72	1.6235	1.2874	0.2020 ± 0.0102	0.2708 ± 0.0142	0.1993 ± 0.0031
G	228	70.37	14.16	1.7037	1.3105	0.2131 ± 0.0101	0.2969 ± 0.0156	0.2068 ± 0.0031
H	235	72.53	9.70	1.7253	1.2971	0.2039 ± 0.0099	0.2899 ± 0.0152	0.1997 ± 0.0029
I	283	87.35	9.10	1.8735	1.3707	0.2474 ± 0.0097	0.3571 ± 0.0187	0.2330 ± 0.0030
J	224	69.14	10.67	1.6914	1.3384	0.2396 ± 0.0100	0.3140 ± 0.0165	0.2271 ± 0.0033
K	239	73.77	9.17	1.7377	1.3588	0.2360 ± 0.0103	0.3282 ± 0.0172	0.2278 ± 0.0028
L	244	75.31	11.14	1.7531	1.3622	0.2501 ± 0.0099	0.3393 ± 0.0178	0.2404 ± 0.0031
M	266	82.1	9.72	1.8210	1.3731	0.2558 ± 0.0094	0.3592 ± 0.0188	0.2407 ± 0.0031
N	237	73.15	12.01	1.7315	1.3378	0.2288 ± 0.0101	0.3188 ± 0.0167	0.2250 ± 0.0029
O	202	62.35	12.22	1.6235	1.3037	0.2079 ± 0.0105	0.2757 ± 0.0145	0.2095 ± 0.0031
Total	324	100.00	10.73	2.0000	1.4433	0.2986 ± 0.0097	0.4291 ± 0.0100	0.2895 ± 0.0008
Mean	251	77.15	10.73	1.7747	1.3653	0.2465 ± 0.0099	0.3407 ± 0.0179	0.2362 ± 0.0030

**Table 3:** Analysis of molecular variance for *I. pseudotinctoria.* Degrees of freedom (*D.f.*), sum of squares (SS), genetic differentiation (*F_ST_*).

Group	Source of Variance	*D.f.*	Sum of Squares	Variance Components	Percentage of Variance (%)	*F_ST_*
Two clusters (lowland vs. highland)	Between Clusters	1	1120.049	11.1906	8.98	*F_CT_* = 0.0898
	Among acc. within Cluster	13	3269.13	8.74707	16.44	*F_SC_* = 0.1801
	Within accessions	349	13844.89	39.67018	74.57	*F_ST_* = 0.2543
	Total	363	18234.07	53.19614		
All accessions	Among accessions	14	4389.178	11.29686	22.17	*F_ST_* = 0.2217
	Within accessions	349	13,844.89	39.67018	77.83	
	Total	363	18,234.07	50.96705		

**Table 4:** Comparison of genetic differentiation for *I. pseudotinctoria* using AFLP markers. Total gene diversity (*H_T_*), average gene diversity within accessions (*H_S_*), genetic differentiation coefficient (*G_ST_*, *G_ST_* = (*H_T_* − *H_S_*)/*H_T_*), total Shannon information index (*I_sp_*), average Shannon information index within accessions (*I_pop_*), Shannon’s coefficient of genetic differentiation (*G’_ST_*, *G’_ST_* = (*I_sp_* − *I_pop_*)/*I_sp_*), gene flow (*N_m_*) from *G_ST_* (*N_m_* = (1 − *G_ST_*)/4*G_ST_*). Significance level of LSD at 0.05.

Variable	All Accessions	Lowland Cluster	Highland Cluster
*H_T_*	0.2986	0.3020	0.2384
*H_S_*	0.2465	0.2641a	0.2142b
*G_ST_*	0.1746	0.1255	0.1015
*I_sp_*	0.4291	0.4406	0.3796
*I_pop_*	0.3407	0.3618a	0.3223b
*G'_ST_*	0.2060	0.1789	0.1511
*F_ST_*	0.2217	0.2124	0.1491
*N_m_*	1.1819	1.7421	2.2128

**Table 5:** Wright’s F-statistics calculated for accessions of *I. pseudotinctoria* under different models based on a Bayesian approach. Bayesian genetic differentiation (*θ*^B^), inbreeding coefficient (*f*), standard deviation (SD), and deviance information criterion (DIC).

Model	*θ*^B^			*f*			DIC
	Mean ± SD	2.50%	97.50%	Mean ± SD	2.50%	97.50%	
full model	0.1899 ± 0.0040	0.1397	0.2094	0.0169 ± 0.0250	0.0140	0.1279	19,432.7
*f* = 0 model	0.1844 ± 0.0022	0.1388	0.1941	0	-	-	19,375.6
*θ*^B^ = 0 model	0	-	-	0.3587 ± 0.2889	0.0316	0.2821	39,248.4
free model	0.1997 ± 0.0123	0.1645	0.2103	0.3987 ± 0.2889	0.0254	0.9721	20,836.1

**Table 6:** The pairwise values of *F_ST_* (below diagonal) and *N_m_* (above diagonal) of *I. pseudotinctoria* accessions.

Accession	acc.A	acc.B	acc.C	acc.D	acc.E	acc.F	acc.G	acc.H	acc.I	acc.J	acc.K	acc.L	acc.M	acc.N	acc.O
acc.A	-	3.0701	2.0691	1.5629	0.9053	0.6658	0.6973	0.6995	1.0474	0.9761	1.0126	1.0353	1.1259	0.8398	0.8989
acc.B	0.0753	-	3.9659	2.4152	1.3125	0.9144	1.0294	1.0742	1.5964	1.4335	1.4507	1.4909	1.5294	1.0714	1.0777
acc.C	0.1078	0.0593	-	9.4025	1.9430	1.3734	1.5460	1.5682	2.6981	2.5749	2.4852	2.4942	2.3705	1.3154	1.2120
acc.D	0.1379	0.0938	0.0259	-	3.2319	1.5909	1.8868	1.8633	3.3784	2.1355	1.9742	2.0104	3.4050	1.7580	1.4484
acc.E	0.2164	0.1600	0.1140	0.0718	-	4.1670	4.4316	2.1562	3.0833	1.7792	1.6790	1.4837	1.9108	1.1312	1.0190
acc.F	0.2730	0.2147	0.154	0.1358	0.0566	-	8.3411	2.2550	2.4067	1.6746	1.4438	1.2077	1.2524	0.7835	0.7472
acc.G	0.2639	0.1954	0.1392	0.1170	0.0534	0.0291	-	7.6364	4.6138	2.0289	1.8579	1.5306	1.5815	0.9037	0.8116
acc.H	0.2633	0.1888	0.1375	0.1183	0.1039	0.0998	0.0317	-	7.1029	1.9683	1.7629	1.4013	1.5499	0.8916	0.8125
acc.I	0.1927	0.1354	0.0848	0.0689	0.075	0.0941	0.0514	0.0340	-	5.5370	4.4316	2.8828	2.9842	1.3713	1.2232
acc.J	0.2039	0.1485	0.0885	0.1048	0.1232	0.1299	0.1097	0.1127	0.0432	-	33.5338	8.2246	2.0563	1.0700	0.9938
acc.K	0.1980	0.1470	0.0914	0.1124	0.1296	0.1476	0.1186	0.1242	0.0534	0.0074	-	11.7117	1.9565	1.0353	0.9416
acc.L	0.1945	0.1436	0.0911	0.1106	0.1442	0.1715	0.1404	0.1514	0.0798	0.0295	0.0209	-	1.8941	1.0321	0.9166
acc.M	0.1817	0.1405	0.0954	0.0684	0.1157	0.1664	0.1365	0.1389	0.0773	0.1084	0.1133	0.1166	-	8.7106	3.5321
acc.N	0.2294	0.1892	0.1597	0.1245	0.1810	0.2419	0.2167	0.2190	0.1542	0.1894	0.1945	0.1950	0.0279	-	4.6520
acc.O	0.2176	0.1883	0.1710	0.1472	0.1970	0.2507	0.2355	0.2353	0.1697	0.2010	0.2098	0.2143	0.0661	0.0510	-

**Table 7:** List of the 15 native *Indigofera pseudotinctoria* accessions sampled for the analysis. Accession name (Acc.), number of individuals (Ind.), elevation (Elev.), longitude E (Long.), latitude N (Lat.), annual mean temperature (T_mean_), annual precipitation (P), soil pH value (pH), and soil organic matter (OM).

Acc.	Ind.	Elev.	Longitude	Latitude	T_mean_ (°C)	P (mm)	pH	Soil Type	OM (g/kg)
A	23	379	109°51’21.97”	31°06’55.85”	16.042	1041	7.40	loess soil	6.2
B	26	552	109°55’40.15”	31°03’16.74”	16.142	1029	7.64	loess soil	21.3
C	23	600	109°53’20.59”	30°56’32.75”	14.733	1150	7.65	purple soil	9.4
D	24	640	109°53’49.32”	30°56’34.47”	14.733	1150	7.72	purple soil	18.1
E	30	1002	109°53’49.30”	30°56’34.47”	14.733	1150	7.30	purple soil	14.4
F	21	1612	109°55’2.82”	30°54’50.84”	14.429	1169	5.85	purple soil	66.3
G	21	1533	109°55’0.68”	30°54’51.78”	14.429	1169	7.50	purple soil	9.9
H	24	1444	109°54’32.72”	30°55’11.86”	14.733	1150	7.91	purple soil	45.0
I	25	1336	109°54’17.56”	30°55’27.16”	14.733	1150	5.83	purple soil	13.9
J	19	1212	109°53’50.89”	30°55’39.22”	14.733	1150	7.66	purple soil	15.6
K	29	1071	109°53’17.98”	30°55’23.87”	14.733	1150	7.85	purple soil	10.5
L	26	1018	109°53’45.56”	30°55’56.45”	14.733	1150	7.16	purple soil	10.6
M	25	913	109°53’38.58”	30°55’57.98”	14.733	1150	7.57	purple soil	10.6
N	27	778	109°53’51.79”	30°56’21.01”	14.733	1150	7.53	purple soil	13.4
O	21	311	109°51’13.92”	31°07’3.41”	17.442	960	7.20	loess soil	15.9
